# Laparothorascope

**Published:** 1914-05

**Authors:** 


Laparothorascope. Rosenthal, *Bull. Gen. de Ther.*, May 30, 1913, discusses the method devised by Jacobeus of Stockholm. An opening is made with a large trochar and a cystoscope introduced. Various lesions can be seen.

